# Current Strategy for Targeting Metallo-β-Lactamase with Metal-Ion-Binding Inhibitors

**DOI:** 10.3390/molecules29163944

**Published:** 2024-08-21

**Authors:** Jessica L. Ortega-Balleza, Lenci K. Vázquez-Jiménez, Eyra Ortiz-Pérez, Guadalupe Avalos-Navarro, Alma D. Paz-González, Edgar E. Lara-Ramírez, Gildardo Rivera

**Affiliations:** 1 Laboratorio de Biotecnología Farmacéutica, Centro de Biotecnología Genómica, Instituto Politécnico Nacional, Reynosa 88710, Mexico; jessica_ortega7@hotmail.com (J.L.O.-B.); lenka.18@hotmail.com (L.K.V.-J.); eortizp@ipn.mx (E.O.-P.); apazg@ipn.mx (A.D.P.-G.); elarar@ipn.mx (E.E.L.-R.); 2 Consejo Nacional de Humanidades, Ciencias y Tecnologías (CONAHCYT), Ciudad de México 03940, Mexico; 3 Departamento de Ciencias Médicas y de la Vida, Instituto de Investigación en Genética Molecular, Centro Universitario de la Ciénega, Universidad de Guadalajara, Ocotlán 47810, Mexico; guadalupe.avalos5337@academicos.udg.mx

**Keywords:** antibiotic resistance, metallo-β-lactamases, carbapenemase, metal ion binding, inhibitors, zinc, carbapenems

## Abstract

Currently, antimicrobial resistance (AMR) is a serious health problem in the world, mainly because of the rapid spread of multidrug-resistant (MDR) bacteria. These include bacteria that produce β-lactamases, which confer resistance to β-lactams, the antibiotics with the most prescriptions in the world. Carbapenems are particularly noteworthy because they are considered the ultimate therapeutic option for MDR bacteria. However, this group of antibiotics can also be hydrolyzed by β-lactamases, including metallo-β-lactamases (MBLs), which have one or two zinc ions (Zn^2+^) on the active site and are resistant to common inhibitors of serine β-lactamases, such as clavulanic acid, sulbactam, tazobactam, and avibactam. Therefore, the design of inhibitors against MBLs has been directed toward various compounds, with groups such as nitrogen, thiols, and metal-binding carboxylates, or compounds such as bicyclic boronates that mimic hydrolysis intermediates. Other compounds, such as dipicolinic acid and aspergillomarasmin A, have also been shown to inhibit MBLs by chelating Zn^2+.^ In fact, recent inhibitors are based on Zn^2+^ chelation, which is an important factor in the mechanism of action of most MBL inhibitors. Therefore, in this review, we analyzed the current strategies for the design and mechanism of action of metal-ion-binding inhibitors that combat MDR bacteria.

## 1. Introduction

Human bacterial pathogens with antimicrobial resistance (AMR) are a critical health problem and are considered a global emergency. In recent years, deaths caused by multidrug-resistant (MDR) bacteria have been on the rise, with around 1.27 million deaths reported in 2019, and these were associated with 4.95 million deaths worldwide [1]. It is estimated that AMR will be the leading cause of death worldwide by 2050 [2]. The most known cause of AMR is the inappropriate use of antibiotics for therapeutic and non-therapeutic purposes. However, it should be noted that not only has the undiscriminating use of antimicrobials promoted AMR but horizontal gene transfer (HGT) is also key in the spread of antibiotic resistance genes (ARGs) between bacteria from different environments, which encourages resistance [3], as this provides organisms with traits for greater environmental adaptability and survival owing to the presence of antibiotics in different niches [4], thus reducing therapeutic options and increasing the problem.

HGT is facilitated by mobile genetic elements (MGEs), such as insertion sequences, transposable elements, or integrons, which can be mobilized between bacteria by other MGEs, such as plasmids and integrative conjugative elements (ICEs), which generate its rapid evolution to adapt to its environment [5]. The three major mechanisms of bacterial HGT are conjugation, natural transformation, and transduction (via plasmids and ICEs, extracellular DNA uptake, and bacteriophages, respectively), of which conjugation is the main mechanism for the spread of ARGs [6] and involves contact between the donor (carrying the plasmid and ARG) and the recipient bacteria; a conjugation bridge is synthesized between the donor and the recipient through the conjugative pili, and MGEs (plasmids or ICEs) can be moved from the donor to the recipient through this channel [7].

**Figure 1:**
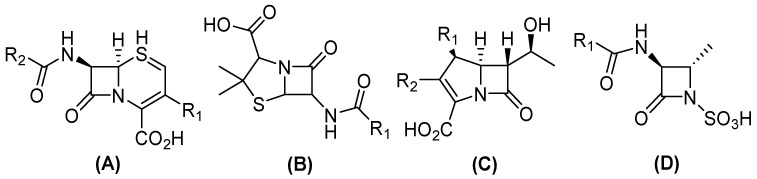
General structure of clinical β-lactam antibiotics. (**A**) Cephalosporines, (**B**) penicillin, (**C**) carbapenems, and (**D**) monobactams.

In this sense, conjugation genes encoding (β) lactamases are one of the most frequently transferred ARGs among Gram-negative bacteria, which contribute to the emergence of β-lactam resistance [8]. Currently, more than thirty-four known β-lactam antibiotics have been approved by the United States Food and Drug Administration (FDA), which can be divided into four groups (Figure 1). Three have a bicyclic structure: (i) penicillin, a β-lactam ring fused with a thiazolidine ring; (ii) cephalosporins, fused with a dihydrothiazine; and (iii) carbapenems, in which the bicyclic system is completed by a pyrroline. Conversely, (iv) monobactams have a monocyclic structure [9,10]. Nevertheless, the distribution of β-lactamase results in the development of resistance to a variety of antibiotics within this family, including carbapenems, contributes to the appearance of pan-resistant bacteria [11].

Furthermore, as of 2019, only six of the thirty-two antibiotics in development are classified as innovators [12]. Owing to the increase in carbapenemase-producing bacteria and their rapid spread, it is important to develop strategies to control the spread and develop new antimicrobial molecules. Therefore, given the rise in infections that are caused by these specific antibiotic-resistant bacteria, this review will focus on metallo-beta-lactamase (MBL) inhibitors that are currently approved for clinical use or in preclinical stages.

In this review, we aimed to summarize metallo-β-lactamase inhibitors based on Zn^2+^ chelation that have been evaluated between 2015 and 2024. For this purpose, a literature search was performed on the PubMed and Google Scholar databases using the keywords metallo-beta-lactamases, MBL, inhibitors, metal ion binding, and Zn^2+^.

## 2. Beta (β)-Lactam and Carbapenem Antibiotics

Beta(β)-lactam antibiotics are the most used drugs around the world, and Gram-negative bacteria have developed resistance to them due to enzymatic inactivation caused by the production of a group of plasmid-encoded beta-lactamase enzymes [13,14,15]. For instance, within this class of antibiotics, carbapenems (meropenem, biapenem, ertapenem, doripenem, and imipenem) are the most important β-lactams and are incorporated in the World Health Organization (WHO) list of essential drugs because they are considered the last therapeutic choice for treating complicated MDR infections when other antibiotics, such as penicillin, have failed, especially members of *Enterobacteriaceae*, due to their capability to adapt to selective pressure, promoting survival [16,17,18].

Unfortunately, carbapenems are becoming less effective with the emergence of new β-lactamases that are not inactivated by available inhibitors. Due to high associated mortality, in 2017, the WHO classified carbapenem-resistant Gram-negative infections—that is, carbapenem-resistant *Enterobacteriaceae* (CRE), carbapenem-resistant *Pseudomonas aeruginosa*, and carbapenem-resistant *Acinetobacter baumannii*—as critically important [19] and in urgent need of new drugs [20]; carbapenem-resistant *Enterobacteriaceae* and third-generation cephalosporins (ceftriaxone, cefpodoxime, ceftazidime, cefdinir, ceftibuten, and others) represent the highest priority for new antibiotic development [21,22].

**Figure 2:**
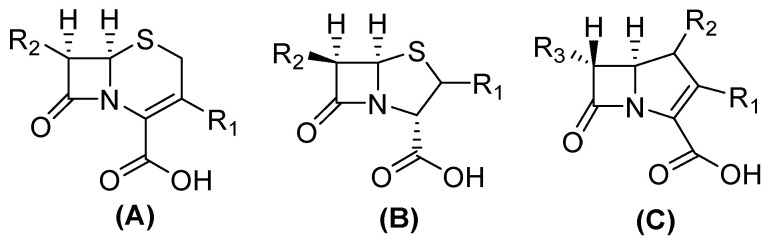
Chemical structure of cephalosporin in cis-configuration (**A**), penicillin in *cis*-configuration (**B**), and carbapenem in *trans*-configuration (**C**).

The structure of carbapenems is a five-membered ring, yet they have a carbon atom at position 1 with a sulfur atom, as in penicillin and cephalosporins, and they have a double bond between C-2 and C-3 of the pentameric ring [23]. The *trans*-configuration (Figure 2) at C5–C6 and the C-6 (R)-hydroxyethyl substituent provide better resistance to the action of β-lactamases versus the *cis*-configuration of penicillin and cephalosporin (Figure 2). Their mechanism of action is the same as that of β-lactams because they are structural analogs of peptidoglycan precursors; thus, they act by interfering with bacterial cell wall biosynthesis by covalently inactivating D, D-transpeptidases, or penicillin-binding proteins (PBPs) through the acylation of a serine active site in these crucial enzymes, leading to the inhibition of peptidoglycan cross-linking in cell walls, resulting in cell lysis and, at the same time, cell death [10,15,24,25].

## 3. Beta(β)-Lactamases

Due to their broad spectrum of activity, ß-lactams are considered one of the most effective antibiotics; however, their excessive and indiscriminate use promotes bacteria survival and has led to the rise of resistant bacteria. This resistance is principally caused by enzymatic inactivation, although mechanisms such as efflux pumps and second mutations that alter the expression and/or function of porins and PBPs are also involved [26,27].

The predominant mechanism observed in clinical isolates is the production of β-lactamases [10]. These enzymes are characterized by the hydrolysis of the β-lactam ring [25]. They are classified into four classes (A, B, C, and D) according to their amino acid sequence homology [13], based on the Ambler classification. Meanwhile, Bush–Jacoby–Medeiros functional categorization considers substrate and inhibitor response profiles and classifies enzymes into three groups (1, 2, and 3) [28,29].

Furthermore, in the Ambler classification classes (A, C, and D), serine β-lactamases use a serine residue to hydrolyze the β-lactam ring. On the other hand, class B, also known as MBLs, possesses Zn^2+^ in the active site, which facilitates the formation of a non-covalent reactive complex with the β-lactam [30,31,32].

## 4. Metallo-Beta-Lactamases (MBLs) as Drug Targets

In this work, we will focus on MBLs, which are characterized by hydrolyzing carbapenems, penicillin, and cephalosporins, but are unable to hydrolyze monobactams [33]. These types of enzymes are usually present in combination with other ESBLs in clinical isolates [28] and account for about 10% of reported β-lactamases, of which only 3% confer resistance exclusively to carbapenems [14,34]. These enzymes use Zn^2+^ as a fundamental cofactor to cut the β-lactam ring and deactivate the antibacterial agents; possess one or two zinc ions (Zn^2+^) in the active site; and are inhibited by chelator agents. In addition, MBL bacteria are resistant to common inhibitors of β-lactamases, such as clavulanic acid, sulbactam, tazobactam, and avibactam [33,34,35].

**Figure 3:**
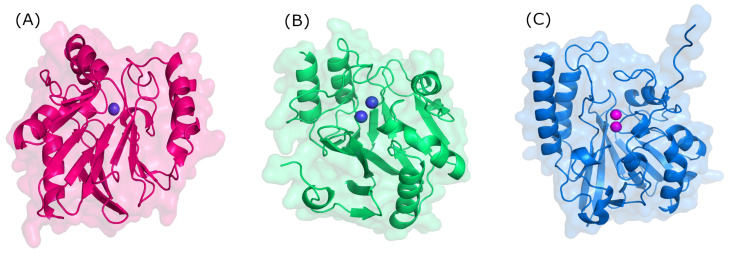
Three-dimensional structure of metallo-β-lactamases. (**A**) B1 (NDM-1, PDB: 3SPU); (**B**) B2 (CphA, PDB: 3F90); and (**C**) B3 (L1, PDB: 1SML).

Structurally, MBLs possess a single, highly conserved globular domain in groups B1, B2, and B3 and present an αβ/βα fold. The active site is situated at the interface between αβ units that employ one or two zinc ions, critical for catalytic activity, and are also flanked by flexible loops (Figure 3) [35,36,37]. Zinc ions are relevant at several stages: substrate positioning on the active site, the activation of nucleophiles, the stabilization of the various species formed, and proton donor positioning [38]. Group B1 and B3 MBLs are characterized by two zinc-binding sites (Zn1 and Zn2), whereas group B2 has only one binding site (Zn2); thus, the only structural feature conserved in all MBLs is the Zn2 site [38,39]. It is believed that the loop region of the active site dictates differences in substrate specificity, although recent structures suggest otherwise [40].

MBL genes are commonly harbored in integrons, transposons, and plasmids and are transferred horizontally, allowing for spread and persistence among bacteria, although they have also been reported to be part of the chromosome [25,33,41].

MBLs in Ambler’s classification are divided into groups B1, B2, and B3 according to the amino acid sequence on the active site, the zinc ligands, zinc stoichiometry, the loop architecture, and the substrate profiles [35] Group B1 is the most extensive and includes the most reported MBLs, such as imipenemase (IMP), integron-encoded Verona MBL (VIM), and New Dheli (NDM) [13]. It should be noted that the amino acid homology between the subclasses is <20% [35,36].

## 5. MBL Inhibitors

The use of β- lactamase inhibitors is one useful strategy for restoring β-lactam activity [42]. However, there are currently no FDA-approved inhibitors of MBLs [43] due to their unsuccessful design, which is a result of either the nature of the zinc ligands and catalytic mechanisms or differences in the active site architecture [44].

**Figure 4:**
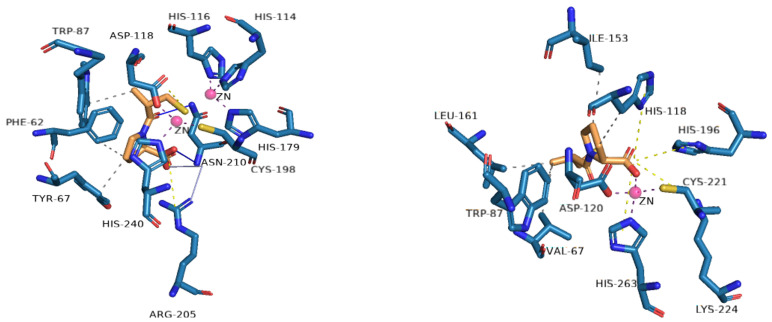
Profile of interactions of zinc on the active site of VIM-2 (PDB: 4C1E) and CphA (2QDS) with D-captopril, an inhibitor.

Inhibitors of MBLs are often grouped according to structural similarities, but they can also be grouped according to their mechanism of inhibition: (1) inhibition by metal ion binding, (2) inhibition by covalent bond formation, (3) allosteric inhibitors, and (4) inhibitors with uncharacterized mechanisms [45]. Most of the reported MBL inhibitors contain a zinc-coordinating group (Figure 4), and numerous metal-binding pharmacophores have been used. The main single or multiple groups reported are the thiol and carboxylate groups [46].

MBL inhibitors bind with zinc, an important factor in their mechanism of action; therefore, this work focuses on metal-ion-binding-mediated inhibition, which, in turn, involves two possible modes of action: *a)* the removal of metal ions (Zn^2+^), wherein the inhibitor removes metal ions from the active site of the enzyme, or *b)* alternatively, the inhibitor can form a ternary complex with the metal ions (Zn^2+^) of the active site of the MBLs and the surrounding protein residues. This process effectively prevents antibiotics from binding to the enzyme, causing the formation of an MBL:Zn^2+^:inhibitor complex [45,47].

**Figure 5:**
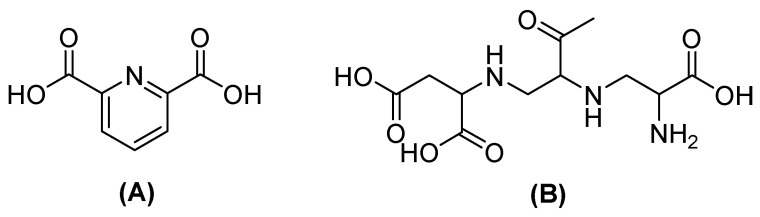
Chemical structure of dipicolinic acid (**A**) and aspergillomarasmin (**B**).

Nevertheless, the co-administration of inhibitors with β-lactam agents has shown efficacy against serine-dependent β-lactamases but not against MBLs. Therefore, the design of MBL inhibitors implicates compounds with carboxylate, nitrogen, and thiol groups that cause metal binding or that simulate hydrolysis intermediates [48]. One of the most promising is captopril, which acts by interfering with nucleophilic hydroxide production due to the thiol group, which binds with Zn^2+^ on the active site in NDM-1; boronic acids also inhibit NDM-1, simulating the transition state of hydrolyzed β-lactamases. Other compounds, such as dipicolinic acid (DPA) or aspergillomarasmin A (AMA) (Figure 5), have also been shown to inhibit MBLs by chelating Zn^2+^ [49].

### 5.1. Metal-Complex-Forming Inhibitors

Several compounds from different groups have been proposed as potential inhibitors against MBLs, with the B1 subclass being the focus. The results show that NDM is more susceptible to inhibition than VIM and IMP.

**Figure 6:**
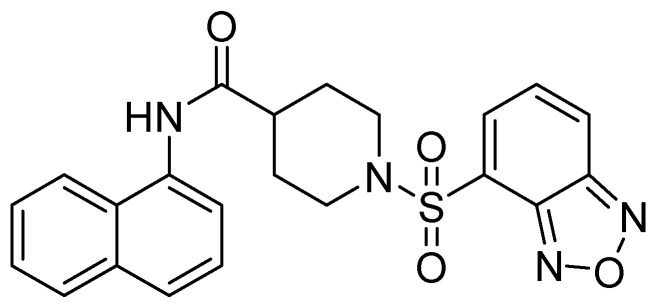
Chemical structure of VNI-41, a potent inhibitor of NDM-1 activity.

In their study, Wang and cols. (2015) identified a potent inhibitor (VNI-41) (Figure 6) that affected NDM-1 activity with an IC_50_ of 29.6 ± 1.3 µM through a broad interaction with the active site (His122, His120, His189, Asn220 His250, and Ile30). The sulfonamide group of compound VNI-41 directly interacts with Zn1, suggesting that compounds with the sulfonamide group could be important inhibitors of MBLs [50].

**Figure 7:**
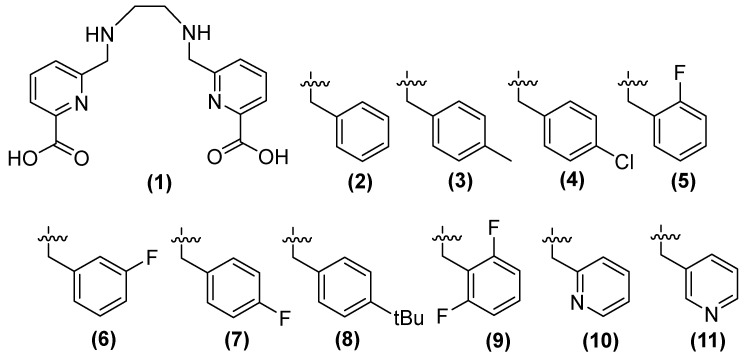
Chemical structure of derivatives of h2dedpa, a metal chelator and inhibitor of NDM-1.

Compound **1** (h2dedpa [1,2-[[6-carboxy-pyridin-2-yl]-methylamino]ethane]) (Figure 7) is a metal chelator and inhibitor of NDM-1. Consequently, Cui and cols. synthesized derivatives (**2**–**11**) of this compound, observing that compounds **9, 7** (substituted aryl), and **11** (substituted aromatic heterocycle) showed the inhibition of NDM-1 at 0.11, 0.06, and 0.46 μM, respectively, when alkyl chain elongation was performed in **9** and **8** and pyridine derivatives were used in **11** [51].

**Figure 8:**
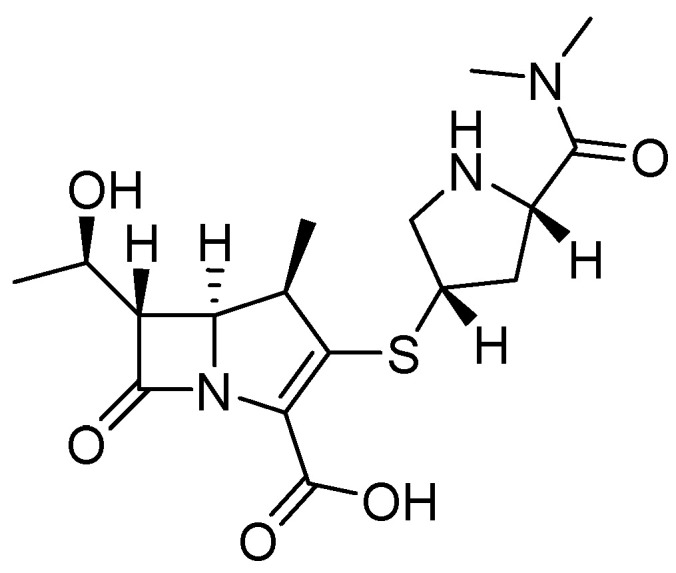
Chemical structure of meropenem (MEM).

The antibacterial activity of meropenem (MEM) (Figure 8) can be restored by compounds **8**, **9**, and **11** against bacteria that produce NDM-1, such as *Klebsiella pneumoniae*, *Klebsiella oxytoca, Enterobacter aerogenes*, *Escherichia coli*, and *Proteus*. Compound **8,** in combination with MEM, reduces the MIC at 1 μg/mL and 0.25 μg/mL in *K. pneumoniae* and *Enterobacter aerogenes,* respectively; **11** shows better results (0.25 μg/mL) in *K. oxytoca*; the same compounds (**8**, **9,** and **11**) show the same MIC (0.25 μg/mL) against *E. coli*, and the best MIC among bacteria has been observed in *P. mirabilis*, with a MIC of 0.06 μg/mL for these three compounds. Other carbapenemases, such as NDM-5, NDM-7, and IMP, have shown less inhibition than NDM-1 [51].

Compound **8** has been observed to exhibit cytotoxic activity. While **9** and **11** have been tested using a computational approach, both compounds have also been demonstrated to inhibit NDM-1 by binding to Zn^2+^. Compound **9** forms a coordinated bound with two zinc ions of NDM-1, while the carbonyl of the other carboxylic acid forms two hydrogen bonds with Lys211 and a fluorine atom in 2,6-difluorobenzene, which binds to Asn220. In compound **11**, the hydroxyl group of one of the carboxylic acids forms a coordinated bond with Zn2 of NDM-1. It has been observed that these compounds increase the efficacy of MEM against *E. coli*,* E. aerogenes*,* K. pneumoniae*, *K. oxytoca*, and *P. mirabilis*, all of which carry the NDM-1 gene [51].

**Figure 9:**
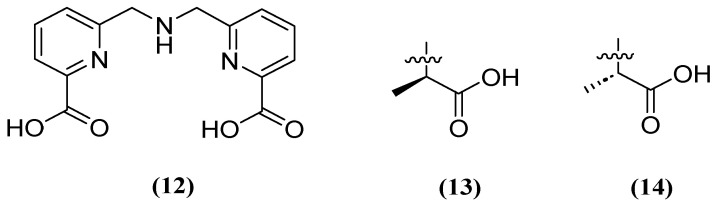
Chemical structure of H_2_dpa (**12**) and derivatives **13** and **14**.

Similarly, H2dpa (**12**) derivatives (Figure 9) containing pentadentate-chelating ligands have shown inhibitory activity against NDM, VIM, and IMP at the molecular, cellular, and in vivo levels. Furthermore, the derivatized compounds designated as **13** and **14** (Figure 9) have been found to be non-competitive and have the ability to restore the activity of MEM against clinical isolates that produce NDM (1.59 μM and 1.57 μM, respectively) and IMP (1.70 μM and 1.54 μM, respectively) [52].

**Figure 10:**
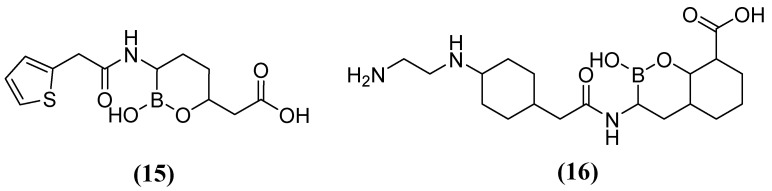
Chemical structure of vaborbactam (**15**) and taniborbactam (**16**).

On the other hand, acyclic boronic acids are known to inhibit nucleophilic enzymes such as serine β-lactamases (SBLs), although cyclic boronates have been observed to exhibit the potent inhibition of MBLs [53]. Vaborbactam **15** (Figure 10), in combination with MEM, has demonstrated the capacity to inhibit SBLs (class A), including KPC carbapenemases. However, it has not exhibited efficacy against MBLs (MIC 32 mg/L) or certain clinically relevant SBLs (MIC 32 mg/L) [54]. Cyclic boronate derivatives such as taniborbactam (TAN) **16** (also known as VNRX-5133) are promising compounds that inhibit serine-dependent β-lactamases and MBLs such as NDM-1, VIM-1, and VIM-2 [55]. TAN has been shown to bind to the metal ion of Zn1 and the conserved amino acid Asn233, located in the VIM-2 active site. In phase I clinical trials, the TAN-CEF (cefepime) combination has been demonstrated to be effective against carbapenemase and extended-spectrum beta-lactamase *Enterobacteriaceae* (ESBL). Specifically, a 4 mg/L TAN dose was required to reduce the cefepime MIC_90_ of the Enterobacterales from 256 to 1 mg/L. Additionally, in *Pseudomonas aeruginosa* cefepime, the MIC_90_ was reduced from 128 to 16 mg/L [56,57,58].

**Figure 11:**
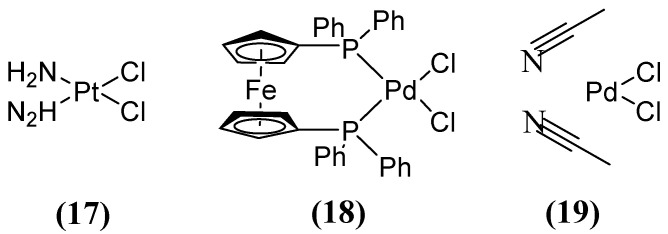
Chemical structures of platinum compound and palladium complexes.

Other compounds, such as those used in chemotherapy against various cancers, like cisplatin (used in approximately 50 % of treated patients) [59], have shown inhibitory activity when combining MEM (Figure 8) with cisplatin **17** or Pd(II)s **18** and **19** (Figure 11). This inhibitory effect has been observed against a broad spectrum of B1 (MIC 8 µg/mL for NDM-1-producing *E. coli*) and B2 MBL subclasses, thereby restoring efficacy against MEM. This demonstrates that these metal complexes are irreversible inhibitors of NDM-1 through a novel mode of inhibition that involves binding to Cys208 and the displacement of the Zn^2+^ ion of the enzyme caused by a Pt(II) ion containing two NH_3_ groups or a Pd(II) ion [60].

Compounds of natural origin have also shown activity as inhibitors, one of which is AMA (Figure 5), a secondary fungal metabolite derived from *Aspergillus versicolor* that can restore MEM antibiotic activity against *E. coli* [61]. This compound alone is not antimicrobial, but in combination with β-lactam antibiotics, it inhibits the catalysis of MBLs. The mechanism of inhibition is based on the sequestration of the Zn^2+^ ion, which spontaneously dissociates from the MBL, but the effect is different for different MBL families and alleles [47].

Previously, the AMA restored effective susceptibility to MEM in *E. coli* and *K. pneumoniae* in strains expressing *bla*_NDM-1_ and *bla*_VIM-2_ (MICs of 4 to 8 μg/mL); NDM-4, NDM-5, NDM-6, NDM-7, IMP-1, IMP-7, and IMP-27 were moderately sensitive (12 to 16 μg/mL). In contrast, subclass B2 and B3 MBLs, CphA2, and AIM-1 exhibit the least susceptibility to AMA inhibition, with MIC values exceeding 64 μg/mL [62]. For example, Sychantha and cols. (2021) evaluated the mechanism of inhibition in vivo and found that AMA leads to the loss of zinc ions (Zn^2+^) from a low-affinity binding site of NDM-1 (8 and 16 μg/mL). This loss contributes to the rapid degradation of NDM-1, which is a crucial factor in the efficacy of AMA as a β-lactam enhancer. However, MBLs with a higher affinity for Zn^2+^, such as NDM-6 or IMP-7, demonstrate increased tolerance to AMA (MICs of 16 and 32 μg/mL) [47].

**Figure 12:**
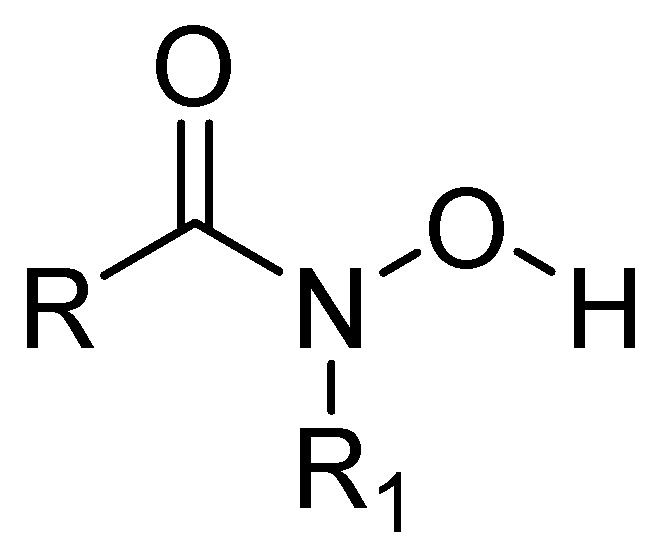
Chemical structure of hydroxamic acid, a metalloprotease inhibitor.

Some metalloprotease inhibitors, such as hydroxamic acid (Figure 12), have a higher capacity to chelate Zn^2+^ and Fe^3+^ and can be used as inhibitors of MBLs [63]. About this matter, the MBL from *Bacillus anthracis* Bla2, which is a member of the B1 subclass of MBLs, contains strongly bound Zn1 and weakly bound Zn2 in its active site. The presence or absence of Zn2 has no significant effect on the catalytic activity of this MBL [64].

**Figure 13:**
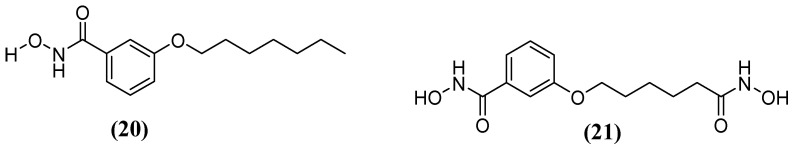
Chemical structure of 3-(heptyloxy)-N-hydroxybenzamide) (**20**) and hydroxamic acid derivative N-hydroxy-3-((6-(hydroxyamino)-6-oxohexyl)oxy)benzamide (**21**).

Kim and cols. (2016) synthetized hydroxamic acid derivatives, including 3-(heptyloxy)-N-hydroxybenzamide) (**20**) and N-hydroxy-3-((6-(hydroxyamino)-6-oxohexyl)oxy)benzamide (**21**) (Figure 13). They did so to evaluate these derivatives against Bla2. The compound 3-(heptyloxy)-N-hydroxybenzamide) was unable to inhibit Bla2, whereas the compound N-hydroxy-3-((6-(hydroxyamino)-6-oxohexyl)oxy) benzamide revealed a competitive reversible inhibition, obtaining a Ki value of 0.18 ± 0.06 μM. Molecular docking analysis indicated that the affinity of the inhibitor compound for the active site of Bla2 is due to coordination with Zn1, with a binding energy of −7.8 kcal/mol. A stable complex was formed between Zn1 and the amino acids His117, Cys197, and His178. The N-hydroxy-3-((6-(hydroxyamino)-6-oxohexyl)oxy) benzamide compound has dihydroxamic acids on both sides, which play a crucial role in its binding affinity. This is in contrast to monohydroxamic acid, which is unable to inhibit Bla2 [65].

**Figure 14:**
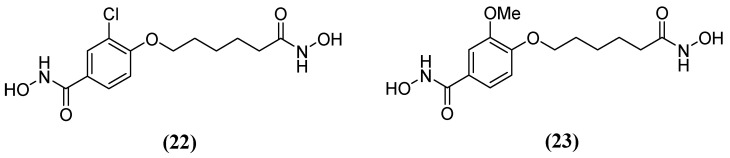
Chemical structure of hydroxamic acid derivatives: (**22**) 3-chloro-N-hydroxy-4-(7-(hydroxyamino)-7-oxoheptyl)benzamide and (**23**) N-hydroxy-4-(7-(hydroxyamino)-7-oxoheptyl)-3-methoxybenzamide.

Hydroxamic acid derivatives 3-chloro-N-hydroxy-4-(7-(hydroxyamino)-7-oxoheptyl) benzamide **22** and N-hydroxy-4-(7-(hydroxyamino)-7-oxoheptyl)-3-methoxybenzamide **23** (Figure 14) have also been tested against Bla2. The results showed that the hydroxamate group is attached to the aromatic end and binds both Zn^2+^ ions during molecular docking. Additionally, the hydrophobic nature of the active site residues Phe63 and Val68 seems to create a more non-polar environment that favors the aromatic end of the compounds. Several potential hydrogen bonds have been identified between these compounds and active site residues. In one study, Lys200, Ser201, and His239 forming a potential hydrogen bond with a hydroxamate group attached to the aliphatic end—referred to as the aliphatic hydroxamate group—with IC_50_ values of 20.0 ± 5.0 µM and 14.9 ± 9.8 µM, were obtained in inhibition assays for **22** and **23**, respectively [64], as well as Asn209 forming a potential hydrogen bond with the aromatic hydroxamate group.

**Figure 15:**
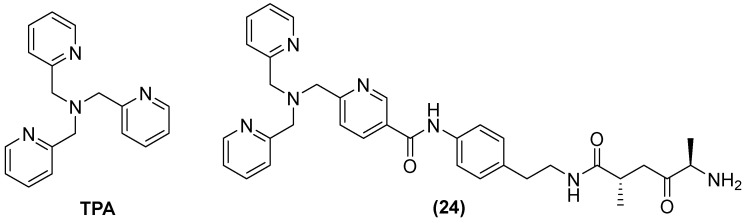
Chemical structure of tris-picolylamine and its derivative, compound **24**.

Tris-picolylamine (TPA) (Figure 15) alone has also shown re-sensitization in clinical isolates of *K. pneumoniae*-producing NDM-1 and *P. aeruginosa*-producing VIM-2, with MIC reductions from 32 to 64 mg/L and 0.125 to 1 mg/L, but toxic effects against the human liver cancer cell line HepG2 were also observed. Therefore, modifications of its structure have also been made by observing the bactericidal activity of MEM in *P. aeruginosa* and *K. pneumoniae* expressing VIM-2 and NDM-1, respectively. The compound designated as **24** reduced the MIC concentration of MEM from 32 to 256 µg/mL at 50 μM in all clinical isolates expressing MBLs [66].

**Figure 16:**
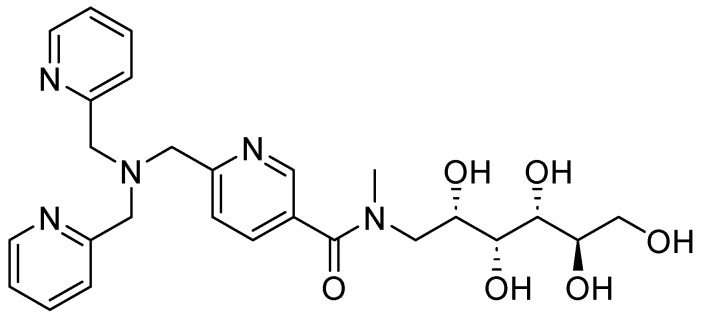
Chemical structure of compound **25**, a zinc chelator.

Other derivatives of TPA have also shown promising results; compound **25** (Figure 16), also a zinc chelator, can restore the bactericidal effect of MEM and in vitro clinical susceptibility to carbapenems by >98%, achieving MICs of ≤2 mg/L in *Enterobacteriaceae*-producing MBLs expressing the enzyme variants NDM, VIM, and IMP. When tested with other carbapenemic agents, such as doripenem and imipenem, **25** reduced the MIC to susceptible levels by >99%. However, this compound has shown favorable aromatic interactions involving Phe61, Tyr67, Tyr224, and Arg228 cation–π interactions with Arg228 between **25** and the active site of VIM-2 in NDM-1. The stacking of aromatic side chains likely results in greater hydrophobic and cation–π interactions between **25** and the VIM-2 active site compared with those involving the equivalent residues in Met61, Val67, Lys224, and Ala228 in NDM-1, indicating that NDM-1 may exhibit reduced enzyme-inhibitor interactions and, consequently, weaker binding. Furthermore, in vivo models at accumulative doses of up to 128 mg/kg, no acute toxicity has been observed [67]. Also, the activity of NDM-1 inhibitors for other metallo-β-lactamases could be influenced by assay conditions, as well as the characteristics of each protein; for this reason, it is important to perform more studies on the mechanisms of action and toxicity [68]. Likewise, it has been noted that potent inhibitors of NDM-1 and other representative MBLs have little inhibition of human zinc-binding enzymes. Further, pharmacokinetic analysis can develop potent β-lactamases using molecular docking and in silico through more studies. However, there is no information on toxicity in this regard.

**Figure 17:**
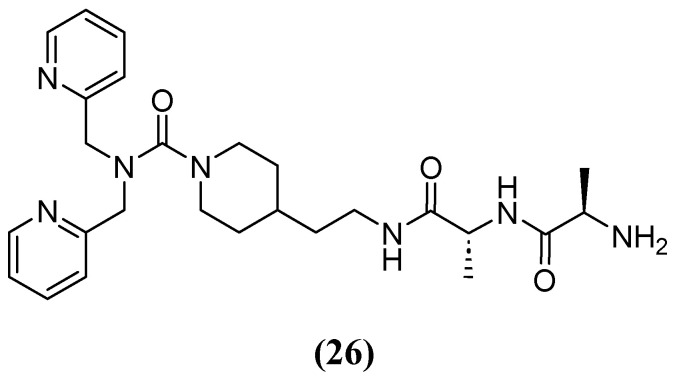
Chemical structure of compound **26**, a derivate of dipicolylamine.

Most chelators are not zinc-specific, but dipicolylamine derivatives have a higher affinity for zinc. Consequently, a series of bivalent hybrids were constructed, consisting of a lipophilic chelator selective for zinc (dipicolylamine) attached to a connector with selective affinity for the bacterial cell structure (D-Ala-D-Ala), with the connector varying in each hybrid. Compound **26** (Figure 17), which has an ethylpiperidine moiety, had the best synergistic effect in combination with MEM, reducing the MIC of MEM from 32 and 64 mg/L to 2 and 1–2 mg/L against clinical isolates producing VIM-2 and NDM-1, respectively. The IC_50_ of **26** against VIM-2 was 9.8 and 2.2 µM at 5 and 20 min, respectively [69].

**Figure 18:**
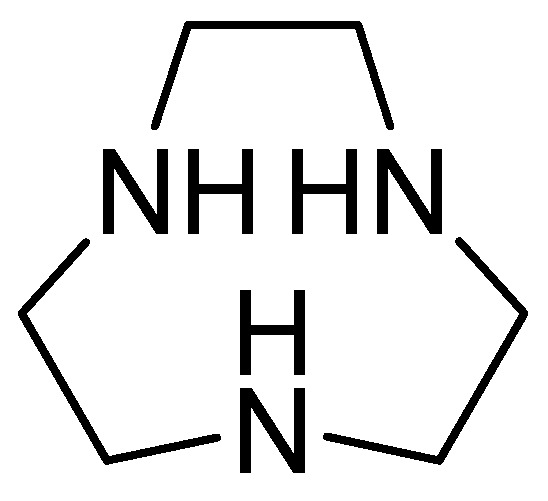
Chemical structure of 1,4,7-triazacyclononane.

1,4,7-triazacyclononane (Figure 18) is derived from cyclononane by substituting three intermediate CH_2_ groups with NH groups and is characterized by a low molecular weight. Its inhibitory activity against MBLs has been observed after joint evaluation with MEM against carbapenem-resistant *Enterobacteriaceae*, obtaining a MIC value = 0.03 mg/L. Furthermore, the combination of MEM with 1,4,7-triazacyclononane causes a significant decrease in the colony-forming units (CFUs) per milliliter. Additionally, kill-time kinetics have shown that this combination causes a 103-fold reduction in the number of CFUs per mL at 4 h in *E. coli* NDM-1, *E. coli* VIM-2, and *Enterobacter cloacae* IMP-1. The inhibitor’s effect has been observed against two key enzymes, NDM-1 and VIM-2, using the new compound, with binding energies of −39.163 kcal/mol and −41.2199 kcal/mol, respectively. Furthermore, the enzyme–ligand complex remains stable. His250 and Asp124 contribute to the hydrogen-bonding capacity, while His122, Asn220, and Trp93 cover the hydrophobic pocket of the active site [70].

**Figure 19:**
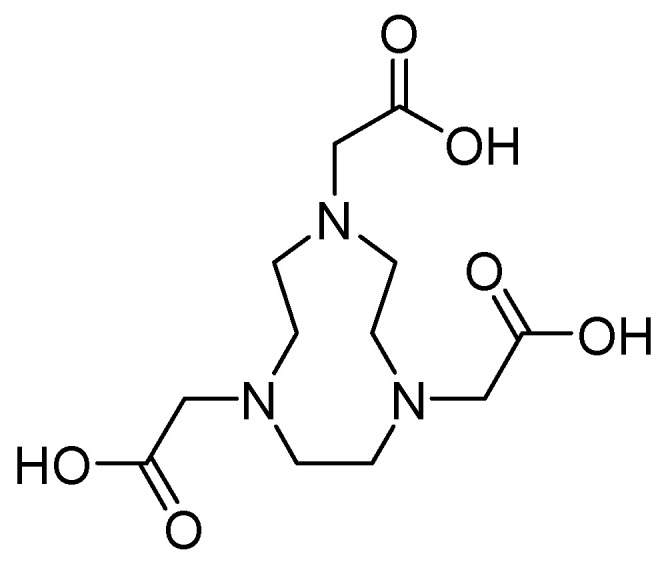
Chemical structure of 1,4,7-triazacyclononane-1,4,7-triacetic acid (compound **27**).

Metal chelators such as 1,4,7-triazacyclononane-1,4,7-triacetic acid **27** (Figure 19) demonstrate a high affinity for zinc. However, when used at concentrations of up to 64 mg/L, they do not inhibit this process. In contrast, the efficacy of MEM has been restored in combination with this antibiotic at a concentration of 0.06 mg/L for *E. coli* IMP-1 and *E. cloacae* NDM-1 and 0.125 mg/L for *K. pneumoniae* NDM-1 and *E. coli* NDM-1 [71].

### 5.2. Formation of Ternary Complexes

Moreover, the inhibition of MBL activity by Zn^2+^ chelation is not specific. Indeed, numerous studies have documented the emergence of mutant MBLs with higher zinc affinity and augmented antibiotic resistance under conditions of Zn limitation [72]. Therefore, other compounds that directly inhibit Zn^2+^ have been investigated. Inhibitors that form ternary complexes include sulfur-containing compounds, such as d-captopril, which exhibit greater specificity for the active site of MBLs and, thus, are less prone to the same adverse off-target effects observed with metal-chelating inhibitors [73].

**Figure 20:**
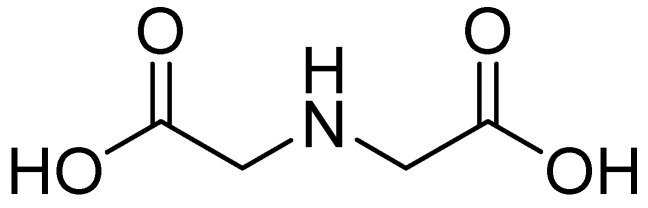
Chemical structure of IDA.

One such compound is iminodiacetic acid (IDA) (Figure 20), an aliphatic derivative of the inhibitor DPA, which is a strong tridentate metal chelator and shares structural features with the chelators AMA and EDTA, the former of which has shown activity against MBLs such as NDM-1.

**Figure 21:**
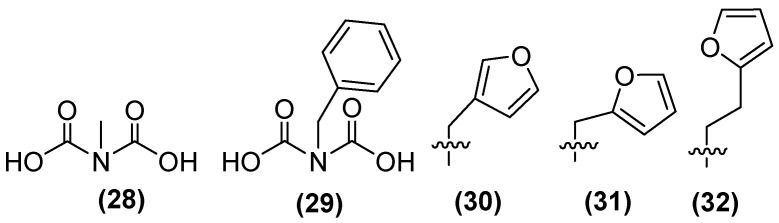
Chemical structure of iminodiacetic acid derivatives as inhibitors potential against NDM-1.

In addition, a small library of compounds has been generated using IDA as the lead structure through fragment-based drug discovery. The first methylated IDA derivative, **28** (Figure 21), exhibited the highest inhibitory activity against NDM-1, reaching 80% inhibition. In a second generation of IDA derivatives, benzylated IDA **29** showed the second highest inhibitory potential (65%). Following the selection of compounds **28** and **29** as scaffolds for inhibitor development, **30** was identified as the most potent inhibitor within this sub-library, exhibiting near complete inhibition against NDM-1 (~99%). A second library containing analogs of **30** with furan and thiophene substituents was also developed. The results showed that the extension from a methyl linker (**31**, IC_50_ = 22 μM) to an ethyl linker (**32**) led to a 2.5-fold enhancement in inhibitory activity, thereby producing the most potent inhibitor of this sub-library (IC_50_ = 8.6 μM) [74].

Compound **32** shows the best results against NDM-1; the ethyl linker allows the furan substituent to interact more favorably with the base of the L3 β-hairpin loop of NDM-1. Ternary complex formation with NDM-1 can also be observed. This, in conjunction with the structural analogy between IDA and the β-lactam ring of the hydrolyzed antibiotic, indicates the possibility of developing analogous transition-state inhibitors of IDA and an alternative AMA scaffold for the creation of NDM-1 inhibitors.

**Figure 22:**
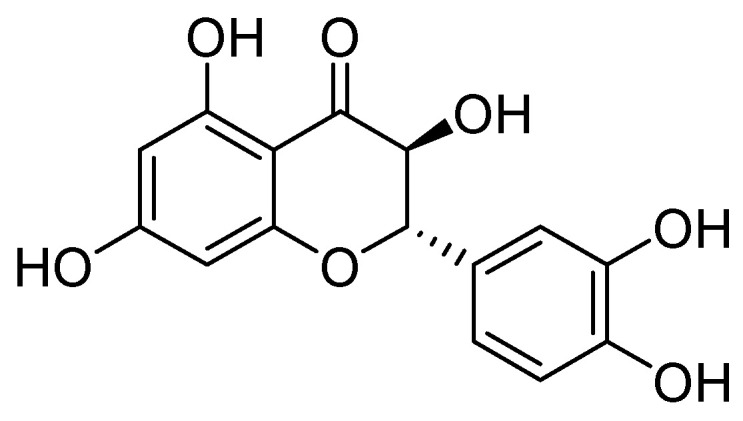
Structure of taxifolin, a promising inhibitor of MBL VIM-2 in *P. aeruginosa*.

On the other hand, taxifolin (Figure 22) is a flavonoid compound, also known as dihydroquercetin, which was originally isolated from the bark of Douglas fir trees. It is commonly found in food items such as onions and olive oil. Additionally, it is a component of various commercial preparations. Its pharmacological activities include antioxidant, anti-inflammatory, and antimicrobial effects [75].

Taxifolin has been found to be an effective inhibitor of VIM-2 in *P. aeruginosa* (MIC = 3.1 µg/mL, Ki = 8 µM). Docking analysis has shown that this compound binds to the VIM-2 binding site in a similar way to quercetin. Taxifolin interacts with the amino acids Asp117 and Arg205 through hydrogen bonding, which contributes to the stabilization of the inhibitor within the active site. Flavonol-derived compounds, such as taxifolin, have the potential to serve as effective inhibitors of MBLs [72].

**Figure 23:**
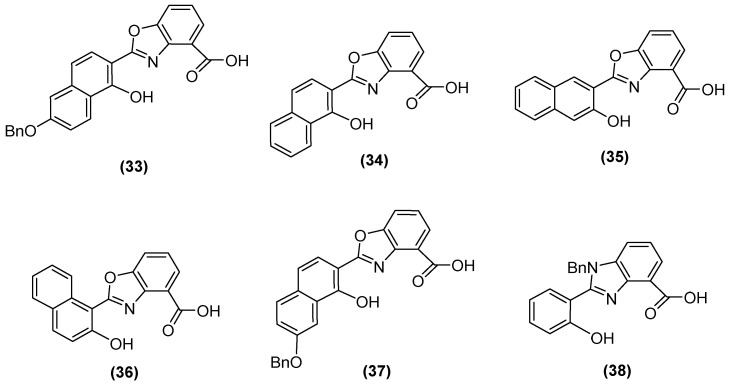
Chemical structure of benzoxazole and benzimidazole derivatives as zinc chelators.

Jackson and cols. (2021) synthesized benzoxazole and benzimidazole derivatives as zinc chelators, observing that six of the compounds exhibited inhibitory activity in *E. coli bla*_NDM-1_ with an IC_50_ value < 10 µM, and compound **33** (Figure 23) had the best activity (IC_50_ = 0.38 µM). The compounds share a similar structural composition, except for the position of the benzyloxy group (compounds **33** and **37**) and an extra benzyl group in compound **38**. In addition, compounds **33**, **34, 37**, and **38** decreased the MIC of MEM (16-fold to 4 μg/mL or less), with compound **33** reaching MIC values < 0.5 μg/mL. It was observed via UV–visible spectroscopy that NDM-1 and this compound form a ternary complex. Molecular docking analysis also showed that compound **33** was coordinated at Zn1 by His120, His122, and His189, while Zn2 was coordinated by Asp124, His250, and Cys208 [73].

In one study, the use of DPA instead of EDTA as a chelator was tested in a disk diffusion analysis observing phenotypic inhibition patterns against MBL IMP-1 and VIM-2 in *P. aeruginosa* and *Acinetobacter* spp., respectively; the results were like the inhibition exerted by EDTA, showing the ability of DPA as a chelator [76].

**Figure 24:**
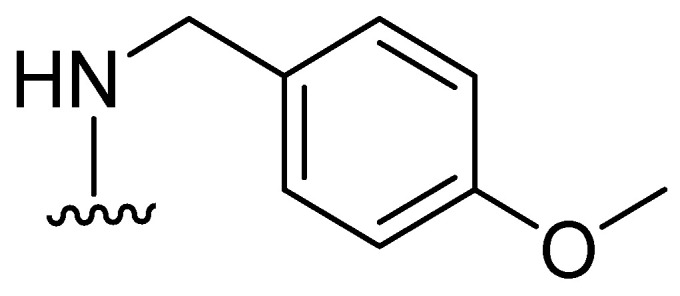
Structure of DPA-derived inhibitor **39**.

Consequently, DPA-derived inhibitors—which are based on DPA and are designed to enhance binding interactions with protein residues proximal to active site Zn^2+^ ions (e.g., Asn220, Gln123, and Lys211)—have been investigated. One of the derivatives, **39** (Figure 24), has demonstrated notable specificity for NDM-1, with an IC_50_ value of 80 nM. In contrast, DPA alone demonstrates a tendency to chelate metal ions from NDM-1. However, **39** forms a ternary NDM-1 complex with Zn^2+^, functioning as a stable inhibitor. The inhibition of IMP-1 and VIM-2 has also been reported. The combination of compound **39** with imipenem demonstrates the restoration of antibiotic susceptibility in clinical isolates of *E. coli* and *K. pneumoniae* harboring NDM-1, as evidenced by a reduction in the MIC (0.5–1 mg/L) [77].

**Figure 25:**
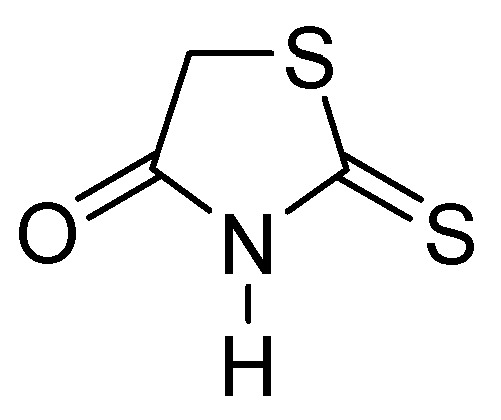
Chemical structure of rhodanine monomer.

Thiazolidinones represent a significant class of heterocyclic compounds with a crucial role in human biological systems, becoming a critical element in the design of novel pharmaceutical agents [78]. Rhodanine (Figure 25) is a subtype of 4-thiazolidinone and has a broad spectrum of biological activity, demonstrating mostly antibacterial, antifungal, antiparasitic, antiviral, antineoplastic, antidiabetic, and anti-inflammatory activity [79].

**Figure 26:**
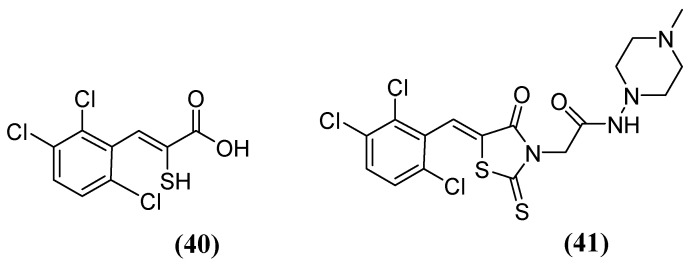
Chemical structure of derivatives **40** and **41**.

The enethiols derived from the hydrolysis of rhodanine inhibit the subclasses of MBLs. Derivative **40** (Figure 26) has been observed to inhibit MBLs by chelating the Zn^2+^ ion from the active site. Notably, an unusual ternary complex is formed between the enzyme and two distinct ligands, **40** and **41** (Figure 26), in the case of VIM-2, which is an unusual occurrence. The most potent inhibition has been observed for **41** against SPM-1, IMP-1, BcII, VIM-2, and NDM-1 (range, 0.02–0.08 μM), while **40** has shown inhibition against IMP-1 (0.09 μM) and VIM-2 (0.06 μM). Interaction studies have shown that thiolate interacts with both Zn^2+^ ions, and carboxylate interacts with Zn2 and Arg233 in VIM-2 [80].

**Figure 27:**
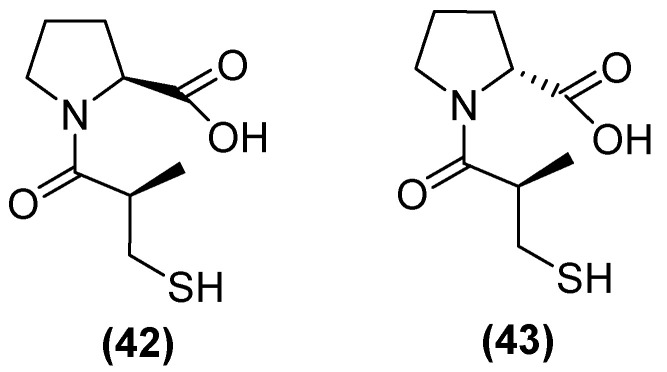
Chemical structure of L-captopril (**42**) and D-captopril (**43**).

On the other hand, thiols—such as captopril, a molecule that contains a thiol and a carboxylate group—are the most widely studied inhibitors. Thiols are potent inhibitors of MBLs due to the ability of sulfur to coordinate zinc. (2 S)-1-((2 S)-2-methyl-3-sulfanylpropanoyl) pyrrolidine-2-carboxylic acid, also known as L-captopril, is a thiol-containing molecule. The D (**42**) and L (**43**) stereoisomers of captopril (Figure 27) are active against MBLs B1 and B3, with their IC_50_ values covering a wide range from 0.072 to >500 μM depending on the captopril stereoisomer and MBL variant [48]. However, thiol-based inhibitors tend to undergo oxidation to the corresponding disulfides, thus losing their zinc-binding and MBL-inhibitory properties [81].

**Figure 28:**
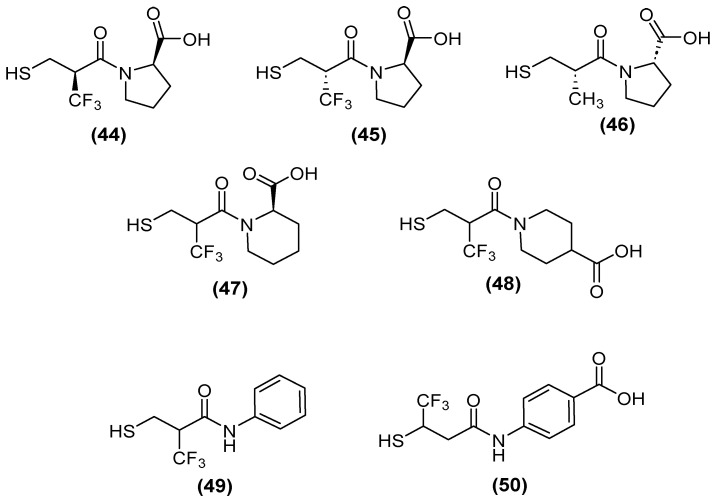
Chemical trifluoromethylated derivatives, analogs of captopril, as NDM1 inhibitors.

Kondratieva and cols. [81] evaluated trifluoromethylated captopril analogs, and the NDM-1 inhibition test again showed that seven of the compounds had IC_50_ values below 10 µM (Figure 28). However, a D-captopril derivative (**45**) showed an IC_50_ value of 0.3 µM and was considered the most active compound. The trifluoromethyl-containing analogs showed the most promising inhibitory activity [81].

Although, several compounds of the trifluoromethylated compounds showed synergistic effects—thus reversing the effect of MEM and reducing the MIC values in NDM-1 (up to 64-fold), VIM-2 (up to 8-fold), and IMP-26 (up to 64-fold) in *E. coli* (Table 1)—both native and trifluoromethyl-substituted inhibitors bind to the two zinc ions of the NDM-1 binding site through their thiol functionality (2.3 Å), and their carbonyl group hydrogen bonds to the side-chain amide of Asn220 (1.6–1.7 Å). An N-H⋯F-C hydrogen bond (2.2 Å) was predicted between the Gln123 amide proton of NDM-1 and the trifluoromethyl group of (+)-5αA [81].

**Figure 29:**
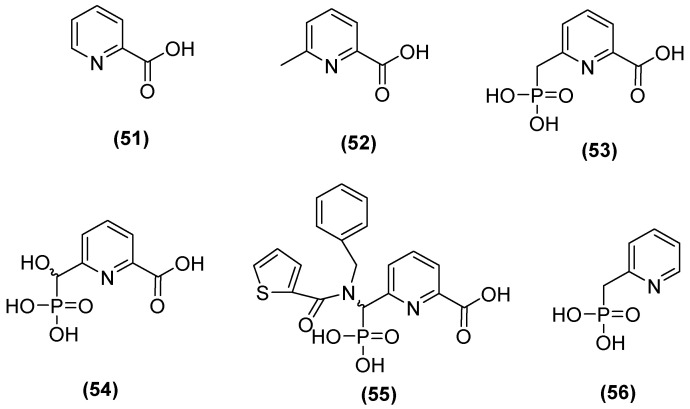
Structure of 6-phosphonomethylpyridine-2-carboxylate (PMPC).

The 6-phosphonomethylpyridine-2-carboxylate (PMPC) compound has also been noted as a potent inhibitor of MBLs such as IMP-1, VIM-2, and NDM-1 in group B1 and L1 in B3. Hinchliffe and cols. [48] synthesized PMPC derivatives (Figure 29) in which the addition of a phosphonomethyl group at C6 of PMCP in 2-picolinic acid (**53**) resulted in significant potency against NDM-1 (IC_50_ = 0.374 μM). The inhibitory activity against NDM-1 (IC_50_ = 0.322 µM) was improved by adding a hydroxyl group to the carbon of the phosphonomethyl group (**54**). However, by adding an additional large hydrophobic substituent to the phosphonomethyl group, it was found that the better compound against all MBLs tested was **55,** with IC_50_ values of 0.306, 0.464, 2.91, and 1.57 µM for NDM-1, VIM-2, IMP-1, and L1, respectively [48].

In addition, in another study, the simplest compound (**53**) was tested to evaluate its ability to enhance the antibacterial activity of MEM against the most clinically relevant subclass B1 MBLs. Co-administration with **53** reduced the MICs of MEM to the susceptible range against all strains tested, except *E. coli* MG1655, which expressed NDM-1 (MIC = 8 mg/L), although 100 mg/L of **53** was required to achieve this. In all cases, the MIC of MEM was reduced by at least 16-fold at 100 mg/mL in **53**. Furthermore, it was determined that **53** binds to the zinc active site of IMP-1 but does not displace the nucleophilic hydroxide; notably, the inhibitory carboxylate group and the pyridine nitrogen atom interacted with the Zn^2+^ site at distances of 2.30 and 2.69 Å on the main chain of the protein (2.70 Å), and the binding was stabilized by interactions between the carboxylate and Lys224 caused by the proximity of a hydrophobic pocket containing Val61, Val67, Trp64, and Phe87 [48].

**Figure 30:**
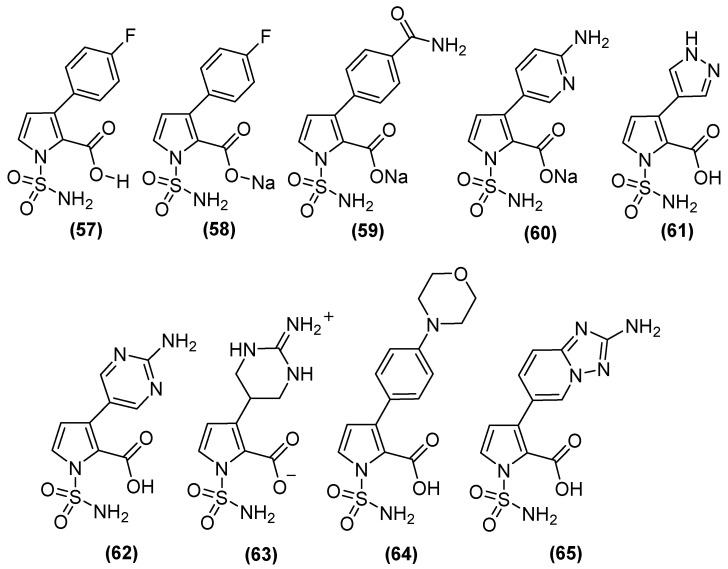
N-sulfamoylpyrrole-2-carboxylate (NSPC) derivatives as potent inhibitors of clinically relevant subclass B1 MBLs.

On the other hand, N-sulfamoylpyrrole-2-carboxylate (NSPC) derivatives (Figure 30) are potent inhibitors of relevant subclass B1 MBLs, including NDM-1, because they can mimic the binding mode of tetrahedral intermediates (boronates). NSPC analogs show inhibitory effects on MBLs (Table 2), of which compound **63** inhibits VIM-1 (pIC_50_ 8.5), which is similar to the bicyclic boronate TAN but less potent against NDM-1. In contrast, analogs such as **57**, **58**, **61,** and **64** show nanomolar potency against NDM-1. NSPC is 150 to >1500 times more potent (pIC_50_ of 7.3 to 9.2) than bicyclic boronates (CB2) or TAN (pIC_50_ values of 6.0 and 5.6, respectively) against IMP-1. Notably, none of the NSPCs can potently inhibit all four MBLs simultaneously. Antimicrobial susceptibility testing has shown that, in NDM-1-producing strains, at a concentration of 8 μg/mL, N-sulfamoylpyrroles reduces the MIC of MEM from 64 to ~0.375 μg/mL [82].

## 6. Conclusions

Given the growing threat of infections caused by MDR bacteria, several compounds and their derivatives have been evaluated in the search for substances capable of inhibiting the most complex group of β-lactamases, the MBLs, for which there are currently no approved inhibitors for therapeutic use, and which are capable of hydrolyzing almost all β-lactams except aztreonam. This review focuses on inhibitors that form ternary complexes with their targets or chelate zinc ions. Zn^2+^ chelation is an interesting strategy with promising results, as seen with AMA inhibitors. However, this mechanism presents a potential risk due to the selectivity toward human metalloenzymes; although, the information regarding this is scarce. Furthermore, the use of chelators as inhibitors may exert evolutionary pressure for the purpose of improving the Zn^2+^-binding affinities of MBLs. Therefore, agents that are highly selective for zinc and weakly bind to ions other than Zn^2+^, such as iron, calcium, manganese, potassium, sodium, and other relevant biological cations, are essential. In addition, boronic acid-based inhibitors such as TAN (VNRX-5133) are the most promising so far, as they can inhibit both serine-dependent b-lactamases and MBLs. The development of new agents against MBLs is a priority, and great efforts are being made to find inhibitors against multiple enzymes that challenge the efficacy of the latest available therapeutic options.

## Figures and Tables

**Table 1:** MICs values of meropenem (MEM) with the best-trifluoromethylated compounds inhibitors against NDM-1, VIM-2, and IMP-26.

Inhibitor	MIC Meropenem (mg/L)		
	MP30-63 *bla*_NDM-1_	MP30-57 *bla*_VIM-2_	MP30-58 *bla*_IMP-26_
No inhibitor control	32	0.5	2
**44**	1	0.06	0.25
**45**	1	0.06	0.5
**46**	4	0.25	4
**49**	1	0.125	2
**50**	0.5	0.125	0.5

**Table 2:** Activity of NSPC derivatives against clinically relevant MBLs.

Inhibitor	pIC_50_			
	VIM-1	NDM-1	VIM-2	IMP-1
Bicyclic boronate	7.1	7.5	8.5	6.0
TAN	8.1	8.0	8.3	5.6
**57**	6.9	8.1	7.7	9.2
**58**	6.9	8.2	7.5	8.9
**59**	7.1	7.9	6.8	8.6
**60**	6.5	7.9	6.7	9
**61**	7.1	8.1	7.8	8.9
**62**	7.4	7.9	7.3	8.2
**63**	8.5	6.5	7.9	7.3
**64**	6.6	8.8	6.8	>9.2
**65**	7.4	7.9	8.2	8.3

## Data Availability

No new data were created or analyzed in this study. Data sharing is not applicable to this article.
